# Calf aversion to hot-iron disbudding

**DOI:** 10.1038/s41598-019-41798-7

**Published:** 2019-03-29

**Authors:** Thomas Ede, Benjamin Lecorps, Marina A. G. von Keyserlingk, Daniel M. Weary

**Affiliations:** Animal Welfare Program, Faculty of Land and Food Systems, University of British Columbia, Vancouver, B.C. Canada

## Abstract

Dairy calves are routinely disbudded by cauterization with a hot iron. To mitigate the intra-operative and initial post-operative pain associated with this procedure some farmers provide calves general and local anesthetics, but it is unknown if the procedure remains aversive. We used a place-conditioning paradigm to assess aversion caused by hot-iron cautery with a local anesthetic compared to a sham procedure. A test area was divided into three equally sized pens: two ‘treatment’ pens with distinct visual cues were connected by a central ‘neutral’ pen. Each calf went through the disbudding procedure and a 6-h recovery period in one treatment pen and the control procedure in the other treatment pen. In three tests (48, 72 and 96 h after the second treatment), calves could freely roam among the pens until they chose to lie down, ending the session. Calves spent less time in either of the treatment pens compared to the central pen. When only comparing the two treatment pen, calves spent less time in the disbudding pen, especially during the first test. Calves were also less likely to lie down in the pen associated with the disbudding procedure. We conclude that even with the use of a local anesthetic, hot-iron disbudding is salient and aversive for calves, indicating the need to refine or avoid the procedure.

## Introduction

Horned cows can be a safety concern for pen mates and their handlers, so the developing horn buds of dairy calves are typically removed via hot-iron cauterization (known as “disbudding”, or “dehorning” when done at a later age)^1^. This procedure is painful^2,3^ and methods to reduce or prevent this pain have gathered considerable scientific interest^4–6^. The procedure can be refined by reducing stress due to handling (using a sedative such as xylazine to facilitate handling and provide weak analgesia) and by treating the intra-operative pain (using local anesthetics such as lidocaine to numb nerves serving the affected tissues^7^). Post-operative pain can also be partly mitigated with analgesics such as nonsteroidal anti-inflammatory drugs (NSAIDs)^8,9^, but these are rarely provided on dairy farms^10–12^ and are unlikely to relieve all the pain associated with the procedure^13^.

Previous work on pain associated with disbudding has primarily focused on physiological measures, especially the cortisol response: a recent meta-analysis on cautery disbudding reported that 19 out of 21 studies included cortisol measures^14^. However, measures related to the hypothalamic-pituitary-adrenal (HPA) axis likely reflect arousal rather than emotional valence (i.e. whether something is experienced as positive or negative)^15^, a key concern for animal welfare^16,17^. For example, stallions exhibit increased secretion of cortisol in response to restraint (negative event) but also in response to sexual stimulation (positive event)^18^.

Also commonly used are “pain-related” behaviors such as ear flicks, head shakes, or head rubs^9,19,20^. Such responses can be informative regarding the acute sensory-discriminative aspect of the procedure (i.e. nociception), but similar behaviors are sometimes also expressed by anesthetized or decerebrate animals^21–23^ suggesting some difficulty in drawing strong inferences regarding the affective-motivational component of the experience^24^.

Finally, more complex spontaneous behaviors such as feed consumption^25^, movement^26^ or play^27^ have also been used. Such behaviors are usually related to activity, and their reduction following disbudding can be interpreted as an attempt to avoid stimulation of the painful area.

To assess the emotional dimension of pain, researchers are increasingly encouraged to include learnt responses, for example using conditioning paradigms^13,28,29^. More specifically, conditioned place avoidance paradigms allow inferences regarding whether an event was experienced negatively. Such paradigms rely on the animal developing an association between a specific environment and a negative experience. For example, rats will avoid an environment associated with the ingestion of an emetic agent^30^ and zebrafish will avoid the side of a tank associated with anesthetic agents^31^. Conditioned place avoidance has also been used to specifically assess pain. For instance, Sufka^32^ showed that rats avoid an environment where they experienced inflammatory pain, but show a reduced avoidance when the pain was mitigated with an analgesic. To our knowledge, place conditioning has not yet been applied to evaluate pain in cattle.

Our aim was to use a place conditioning paradigm to assess aversion associated with hot-iron cautery (when mitigated with a sedative and local anesthesia) versus aversion to a sham procedure involving sedation only and no hot-iron cautery. We predicted that calves would show conditioned aversion to the pen where they were disbudded and allowed to recover compared to the pen where they experienced the control procedure.

## Methods

### Ethical statement

The study was approved by the UBC Animal Care Committee (Application A16-0310) and performed in accordance with the guidelines outlined by the Canadian Council of Animal Care^33^.

### Animals and housing

This study was conducted from October to December 2017 at the University of British Columbia Dairy and Education Centre in Agassiz, British Columbia. Thirteen female Holstein calves with a birthweight of (mean ± SD) 37.2 ± 5.0 kg were randomly enrolled at (mean ± SD) 35 ± 14 d of age. Four animals were excluded from the experiment (cf *statistical analysis*).

Animals were individually housed in sawdust bedded pens (2.1 × 1.2 m) for the first 5 d. At day 6, calves were paired in a double sized pen (2.1 × 2.4 m) with another calf of approximately the same age and weight, both calves of the pairs were enrolled. Calves were fed 4 L of whole milk twice a day using a nipple bottle (at 0800 and 1600 h) and had *ad libitum* access to hay, grain and water in their housing pen.

### Apparatus

**Figure 1:**
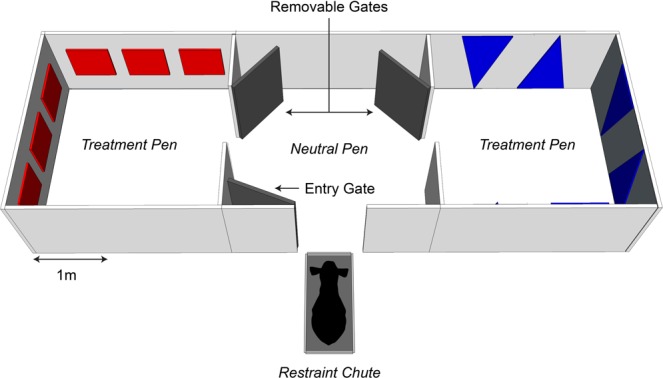
Experimental apparatus. During Treatment sessions, calves were locked in their assigned treatment pen for 6 hours. During pre-treatment exposure and test sessions, removable gates were taken out to allow calves to freely roam between pens until they chose to lie down.

The apparatus was a 2.1 × 6.0 m plywood pen divided in three equal areas (2.1 × 2.0 m; Fig. 1). Two ‘treatment’ pens had colored panels on the walls (either 3 red squares or 2 blue triangles on each wall) and were connected by a ‘neutral’ pen with removable gates. The visual cues (distinct colors, shapes and numbers) were intended to help calves establish an association between pen and treatment. A chute was positioned outside the apparatus, facing the entry gate to restrain the calves before they entered the apparatus.

### Protocol

The experiment consisted of three phases: pre-treatment exposure (one session), treatment (two sessions: control and disbudding) and test (three sessions: 48 h, 72 h and 96 h after the second treatment).

### Pre-treatment exposure

To avoid the potential influence of novelty on place conditioning^34^, calves were pre-exposed to the apparatus for a single session^32^. No additional pre-exposure sessions took place to avoid the weakening of the subsequent conditioning^35^.

Calves were always studied individually. During pre-exposure, at approximately 11:00 h the calf was gently brought from its home pen to the chute in front of the entrance to the apparatus (around 20 m) where she received a 0.25 L milk reward from a nipple bottle. The calf was then let into the apparatus with all compartments accessible. Time spent in each compartment was recorded (calves were considered in the compartment when both front legs were inside) for 15 min. The calf was then let out and brought back to its home pen. Treatment pen assignment was counterbalanced with the preference measured during pre-treatment exposure: half the calves were disbudded in the preferred pen and the other half was only sedated. The pen associated with disbudding (blue triangles or red squares) and order of treatment were assigned in blocks and counterbalanced.

### Treatments (1 and 2)

Twenty-four hours after pre-treatment exposure, the calf was brought from its home pen and injected with xylazine (0.1 mg/kg BW, Rompun, 20 mg/mL, Bayer, Leverkusen, Germany) subcutaneously on the right side of the rump while receiving a 0.25 L milk reward in the chute. The subcutaneous route was chosen as it has been found to be less aversive^36^ but as effective as intra-muscular delivery. The calf was then led into one of the treatment pens (and given the local block, shaved and disbudded if during a disbudding session) and locked into this pen for the next 6 h, such that the calf was able to associate with the pen the recovery from sedation and post-operative pain. The calf was then let out and brought back to its home pen. The next conditioning session took place 48 h later, during which the calf was exposed to the treatment and pen opposite to that used in the previous session.

During disbudding sessions, approximately 10 min after the injection of xylazine in the chute, sedation level was verified as defined in Ede *et al*. (in press). If the calf was recumbent with a noticeable eyeball rotation, she received a local block applied on the corneal nerve by injecting 5 mL per side of lidocaine (Lido-2 [Lidocaine 2%, Epinephrine 1:100,000], Rafter8, Calgary, Canada) with a 0.9 mm × 25 mm needle (8881251782, Covidien, Dublin, Ireland) inserted in the depression between the lateral canthus of the eye and horn bud. Five minutes after the lidocaine injection, the area around the horn buds was shaved and calves were disbudded using a hot iron (X30, 1.3 cm tip, Rhinehart, Spencerville, IN, USA) preheated to approximately 500 °C and applied for approximately 15 s. Although it was not done in this study, we recommend assessing the efficacy the local block prior to disbudding by testing the responsiveness of the calf to a needle prick^37^.

### Tests (1, 2 and 3)

Conditioned-place-aversion testing took place in 3 sessions at 48, 72 and 96 h after the second treatment (at approximately 11:00 h). The test procedure was identical to that during pre-treatment exposure (i.e. free roaming between pens) but lasted 90 min or until the calf lay down (i.e. sternal or lateral recumbency) for at least 1 min (all but one calf lay down within 90 min). Calves were then returned to their home pen.

### Statistical analysis

Using the power.t.test R function^38^, a sample size of 10 animals was recommended for a power of 0.8, significance level of 0.05 and effect size equal to the standard deviation for paired t-tests. Unfortunately, 4 out of the initial 13 calves were excluded from the experiment. One fell sick after the first test (lethargic, rectal temperature of 40.5 °C), two jumped out of the apparatus during the first treatment session and one remained immobile during the pre-treatment exposure. These last three animals may have been particularly sensitive to the test apparatus and the associated social isolation.

Time spent in the pens was analyzed using a linear mixed-effects model using the lme4 R package^39^ testing the fixed effects of treatment received in the pen (Disbudding, Control or Neutral [middle pen]; 2 df), test session (i.e. 1^st^, 2^nd^ or 3^rd^ test; 1df), their interaction (2 df), treatment order (disbudding occurred during the first versus second treatment; 1 df) and the color associated with disbudding (red or blue; 1 df). Calves were considered a random effect, resulting in 81 repeated observations: 3 (time spent in each pen) × 3 (test session) × 9 (calf). Normality and homoscedasticity of residuals were achieved by transforming the time data with a square-root transformation. *P*-values were obtained with Satterthwaite’s approximation using the lmerTest R package^40^. A second similar model only focused on the time spent in the two treatment pens (Disbudding and Control; 1 df). Normality and homoscedasticity of residuals of this second model were also achieved by transforming the time data with a square root transformation. Data available in supplementary material.

The pen in which the calves chose to lay down, in relation to treatment experienced in the pen, was analyzed using a chi-square test.

## Results

### Pre-treatment exposure

Time spent in the pen where the calves would experience disbudding versus time spent in the pen where they would experience the sham procedure were not found to differ during pre-treatment exposure (paired t-test, t_1,8_ = −0.3, *P* = 0.8), indicating that there was no pen bias before calves experienced the treatments.

### Tests

**Figure 2:**
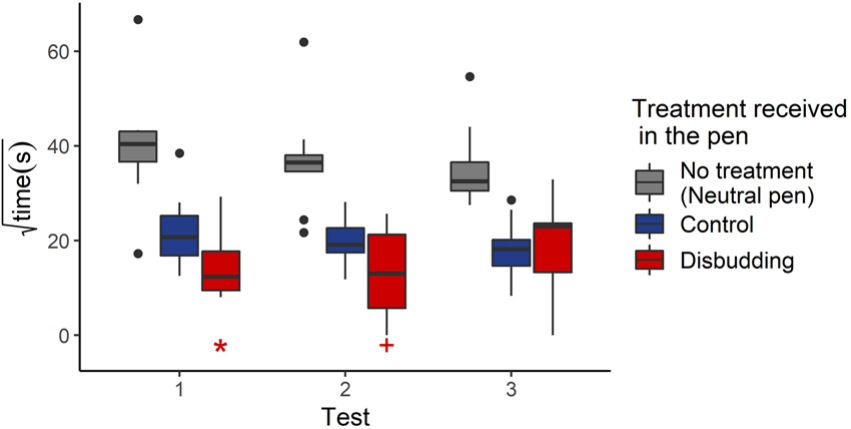
Time (√s) that calves spent in the different pens during test sessions relative to the treatment they received in that pen during treatment sessions (Test 1: 48 h, Test 2: 72 h, Test 3: 96 h after the second treatment). Neutral: No treatment. Control: Sedation alone. Disbudding: Sedation, local anesthesia and hot-iron disbudding. All times spent in treatment pens (control and disbudding) were lower than time spent in the middle pen (P < 0.05). The asterisk (*) represent a significant difference (*P* < 0.05) of the time spent in the disbudding pen compared to time in the control pen. (+) represents a tendency (P = 0.1).

Order of treatment and color of the pen associated with disbudding had no effect on where calves spent their time (t_1,6_ = 0.1, *P* = 0.9; t_1,6_ = 0.6, *P* = 0.6, respectively). Calves spent less time in the treatment pens compared to the middle pen (Control pen: t_2,67_ = −3.3, *P* = 0.001; Disbudding pen: t_2,67_ = −5.4, *P* < 0.001; Fig. 2). Compared to the first session, there was a tendency for the calves to spend more time in the disbudding pen during following sessions (t_2,67_ = 1.7, *P* = 0.1).

When only focusing on time spent in treatment pens (second model), order of treatment and color of the pen associated with disbudding also had no effect on how the calves spent their time (t_1,6_ < 0.1, *P* > 0.9; t_1,6_ = 0.4, *P* = 0.7, respectively). Calves spent less time in the disbudding pen compared to the control pen (t_1,43_ = −2.4, *P* = 0.02). When analyzing test sessions individually with paired t-tests^38^, calves spent significantly less time in the disbudding pen compared to the control pen during the first session (t_1,8_ = −9.8, *P* < 0.001), tended to spend less time in the disbudding pen compared to the control pen during the second session (t_1,8_ = −1.7, *P* = 0.1) and no difference was found during the third session (t_1,8_ = −0.03, *P* > 0.9). See Fig. 2.

**Figure 3:**
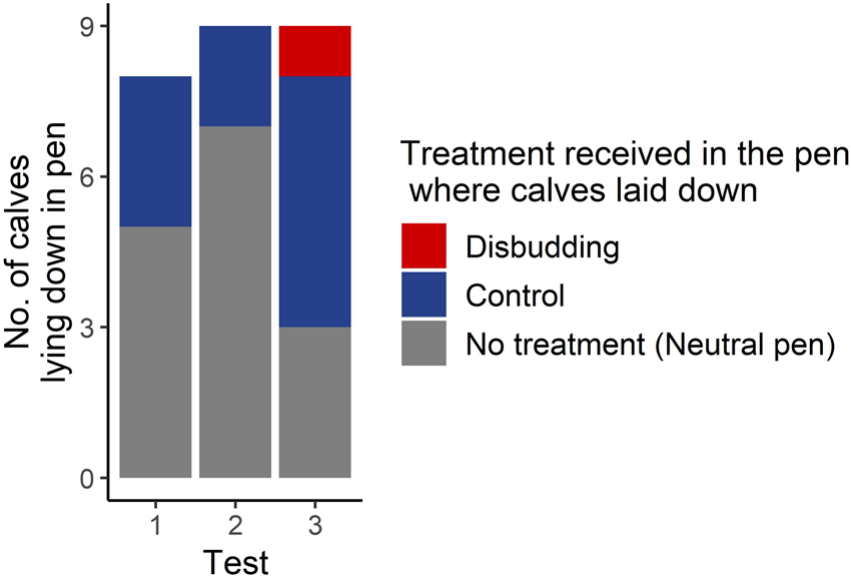
Pen in which calves lay down during test sessions relative to the treatment they received in that pen during treatment sessions (Test 1: 48 h, Test 2: 72 h, Test 3: 96 h after the second treatment). Neutral: No treatment. Control: Sedation alone. Disbudding: Sedation, local anesthesia and hot-iron disbudding. In the first test only 8 of the 9 calves lay down within the 90-min limit.

Calves lay down less frequently in the pen where they had experienced and recovered from hot-iron disbudding (Fig. 3) compared to the other pens. Across all three test sessions, a calf lay down in the pen associated with disbudding just once, compared to 10 times in the pen associated with sedation and 15 in the middle pen (χ² = 11.6, *P* = 0.003).

## Discussion

Calves spent more time (and lay down more often) in the middle pen than in either of the treatment pens, which could indicate that both disbudding and the control procedure were aversive. However, the calf was placed in the middle pen at the start of the test, and the calves had to go through the middle pen to move from one treatment pen to another; both factors could account for the apparent prefence for the middle pen.

When only comparing treatment pens, calves displayed conditioned place avoidance to the pen where they had experienced and recovered from disbudding compared to the control procedure. This avoidance was likely due to calves learning to associate the pen with the pain experienced for the 6 h during and after disbudding. Calves were only provided a single conditioning session in which to associate the pain and the pen, suggesting that this pain was salient to the calves.

Aversion was most apparent during the first test session and waned in following sessions. This pattern was expected given that each test session served as a type of extinction training (as pens were no longer associated with any treatment)^41^.

The place conditioning paradigm used in the current study does not allow us to determine when or what part of the experience was aversive: we can only conclude that it was aversive over the 6 h period. Previous work has shown that the local anesthetic effects of lidocaine persist for approximately 1 to 2 h; after this period plasma cortisol and pain-related behaviors (e.g. head shakes, head rubs and ear flicks) increase for calves that have been disbudded^9,42,43^. The aversive experience is also likely to exceed 6 h: depending on the measure chosen, disbudded calves differ from control calves for a few hours^14^, a few days^26,44^ or up to more than 100 days after the procedure^45^.

To our knowledge, the only previous attempt to assess the emotional impact of disbudding has been through cognitive-bias testing^46,47^; this work found that calves showed a negative judgment bias in the hours after hot-iron disbudding (i.e. calves were more likely to respond negatively to ambiguous cues during a period when they were likely experiencing post-operative pain). Negative judgment biases are consistent with negative emotional states^48,49^ but only allow assessments while the negative state is experienced. The current study shows that a single pairing of disbudding induced fear conditioning (i.e. avoidance of an otherwise neutral environment), providing the strongest evidence to date of the procedure’s negative emotional impact on the calves. Indeed, alpha-2 sedatives like xylazine can cause memory impairments^50^, so the aversion observed in the current study (which is based on how well calves remember the event), might be an underestimation of how negatively this procedure is perceived.

The design of the current study does not enable us to conclude that the aversion was due to pain *per se*; further work using post-operative analgesics would allow for stronger conclusions. However, the current design does allow us to rule out the experience of recovery from sedation as being the reason for the aversion, as animals experienced this recovery in both the treatment and sham conditions. In the treatment condition, calves also experienced lidocaine injections; it is possible that some elements of this procedure may also have been aversive. We did not include cornual injections in the control treatment as we feared animals who had previously been disbudded would have heightened sensitivity to this area compared to animals receiving the control treatment first, resulting in a treatment order bias.

Although we did not find an order of treatment effect, our low sample size might have not been able to detect a carry-over effect: calves who were disbudded during their first conditioning session might have still been experiencing pain 48 h later when exposed to the second conditioning session. We are aware that low sample size is a limitation of this study, but we wished to minimize the number of calves disbudded without post-operative analgesia.

We suggest that place conditioning could be used to investigate the efficacy of post-operative pain control strategies following disbudding (e.g. the use of nonsteroidal anti-inflammatory drugs such as meloxicam or ketoprofen; see^14^, including the effects of drug, dose, route of administration, number of treatments, timing and age of the animal). The results of the current study indicate that place conditioning paradigms may be usefully applied to other species and procedures that have, until now, predominantly been tackled using physiological and pain-related behavioral measures, such as castration in lambs^51^, dogs^52^, piglets^53^ and cattle^54,55^.

## Conclusion

The emotional impact of disbudding is aversive to dairy calves. We recommend the use of effective methods of mitigating this aversion, or that the procedure be discontinued (for example, by breeding for genetically hornless “polled” calves). More generally, we encourage researchers to adopt response measures specifically intended to assess the affective component of painful procedures used on animals.

## Supplementary information

data set

## Data Availability

Data and R code are freely accessible in supplementary materials.
